# Food fortification for bone health in adulthood: a scoping review

**DOI:** 10.1038/ejcn.2016.42

**Published:** 2016-03-30

**Authors:** S J Whiting, W M Kohrt, M P Warren, M I Kraenzlin, J-P Bonjour

**Affiliations:** 1 College of Pharmacy and Nutrition, University of Saskatchewan, Saskatoon, SK, Canada; 2 Department of Medicine, Division of Geriatric Medicine, University of Colorado, Denver, CO, USA; 3 Department of Obstetrics and Gynecology and Medicine, Columbia University Medical Center, New York, NY, USA; 4 Department of Medicine, Division of Endocrinology, Diabetes and Metabolism, University Hospital, Bale, Switzerland; 5 Department of Internal Medicine Specialties, Division of Bone Disease, University Hospitals and Faculty of Medicine, Geneva, Switzerland

## Abstract

Food fortification can deliver essential micronutrients to large population segments without modifications in consumption pattern, suggesting that fortified foods may be formulated for populations at risk for fragility fractures. This scoping review determined the extent to which randomized controlled studies have been carried out to test the impact of fortified foods on bone outcomes, searching PubMed for all studies using the terms ‘fortified AND bone', and ‘fortification AND bone'. Studies were restricted to English language, published between 1996 and June 2015. From 360 articles, 24 studies met the following criteria: human study in adults ⩾18 years (excluding pregnancy or lactation); original study of a fortified food over time, with specific bone outcomes measured pre- and post intervention. Six studies involved adults <50 years; 18 involved adults ⩾50 years. Singly or in combination, 17 studies included calcium and 16 included vitamin D. There were 1 or 2 studies involving either vitamin K, magnesium, iron, zinc, B-vitamins, inulin or isoflavones. For adults <50 years, the four studies involving calcium or vitamin D showed a beneficial effect on bone remodeling. For adults ⩾50 years, *n*=14 provided calcium and/or vitamin D, and there was a significant bone turnover reduction. No consistent effects were reported in studies in which addition of vitamin K, folic acid or isoflavone was assessed. Results from this scoping review indicate that up to now most studies of fortification with bone health have evaluated calcium and/or vitamin D and that these nutrients show beneficial effects on bone remodeling.

## Introduction

Over the last two decades, the impact of nutrition on bone health has been extensively considered, and various diets, foods and nutrients have been studied for their potential effects on bone.^1, 2^ The relation between dairy food products and bone acquisition, as well as maintenance, has been the object of several reports.^3, 4, 5, 6, 7, 8, 9^ The impact on bone health of animal and/or plant protein macronutrients has been a prominent topic in various reports (see for review ref. 10). Among micronutrients, calcium and vitamin D are the two elements that were most frequently studied in randomized controlled trials involving supplements and their effects on various bone outcomes, including fragility fractures.^11, 12, 13, 14, 15^ The role of other micronutrients, vitamin K, magnesium, phosphorus and strontium, has also been evaluated in regard to calcium economy, bone metabolism and resistance to mechanical loading (see for review ref. 16). The effects of potassium and bicarbonate in relation to acid–base balance and bone health have been the subject of several reports.^17, 18, 19, 20, 21^ Other nutrients such as manganese, zinc, iron, isoflavones and soluble fibers have received some attention as to their effects on bone.^22, 23, 24^ Most of the evidence is from studies of supplemental micronutrients.^25^ The European Food Safety Authority has published positive opinions for making a claim that vitamin K^26^ magnesium,^27^ phosphorus,^28^ manganese^29^ and zinc^30^ are beneficial to the general population (Table 1). Presently European Food Safety Authority allows fortification only with those micronutrients that are inadequate in the European diet, that is, only for vitamin K^26^ and magnesium.^27^ However, few actual fortification studies have been carried out with these two micronutrients.

## Food fortification: definition and general aims

Food fortification is the process of adding micronutrients to foods. As defined by the World Health Organization (WHO) and the Food and Agricultural Organization of the United Nations, fortification refers to ‘the practice of deliberately increasing the content of an essential micronutrient, that is, vitamins or minerals in a food, irrespective of whether the nutrients were originally in the food before processing or not, so as to provide a health benefit with minimal risk to health'.^31^ Certain types of fortification are more accurately called enrichment in which micronutrients added to food are those that are lost during processing.^31^ Micronutrient malnutrition is frequent and severe in the developing world; nevertheless, it can also represent a public health problem in more industrialized countries.^32^ Food fortification has the advantage of delivering essential nutrients to large segments of the population without requiring radical changes in food consumption patterns.^32^ Foods used as fortification vehicles vary from country to country, but they generally include cereals and cereal-based products, milk and dairy products, fats and oils, tea and other beverages, and various condiments such as salt, soy sauce and sugar. In practice, the choice of any combination of food vehicle and fortificant is mainly governed by both technological and regulatory factors.^31, 32^

## Importance of food fortification for reducing risk of bone fragility

Bone is a living tissue, and as such all essential nutrients are needed to maintain bone integrity throughout the life cycle. When dietary intakes do not meet needs, nutrient gaps can be filled by means of supplementation and/or fortification. Many studies on the relationship between diet and bone involve supplements because their use facilitates the setting up of randomized clinical trials that compare tablets containing or not the active ingredient. However, dietary surveys in industrialized countries indicate that the prevalence of supplement use in the population can considerably vary, from 40–53% in the USA and Canada^33, 34^ to 9.3–26% in Europe.^35, 36^ A recent systematic review has assessed the efficacy of various micronutrients on bone health in older adults by selecting and summarizing six studies involving supplements, namely calcium with or without vitamin D and vitamin K.^25^ However, in this systematic review of randomized controlled trials,^25^ there is no mention of fortification studies involving these and other micronutrients deemed beneficial to bone. Therefore, it is of interest to determine the impact of food fortification on bone in adulthood, with particular emphasis in postmenopausal women and older adults.

## Scoping review methodology

**Figure 1:**
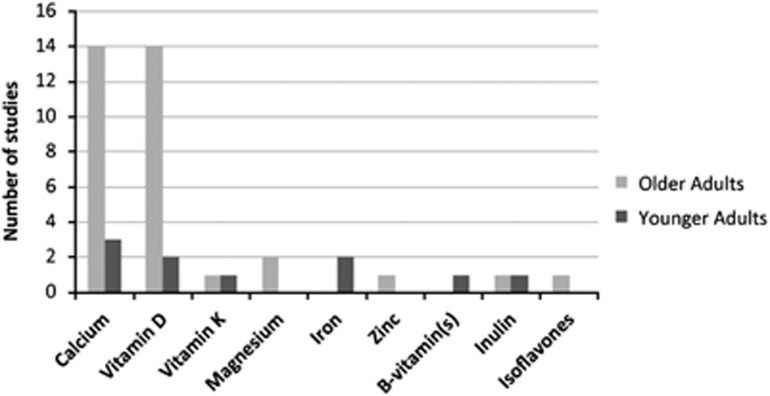
Nutrients added to foods and tested for bone effects: results of a scoping review from 1996 to 2015. Number of included studies distributed according to the added nutrients. Out of 360 articles, 24 studies, 6 and 18 in adults less than and ⩾50 years, respectively, met the predetermined following criteria: human study in adults ⩾18 years; original trial testing fortified foods over time, with specific bone outcomes measured pre- and post intervention.

As assessment of potential size and scope of available literature, we have conducted a scoping review with the aim of determining to what extent studies aimed at measuring the effects of fortified foods on bone have been carried out.^37^ We searched PubMed for all studies using the terms ‘fortified' AND ‘bone', and ‘fortification' AND ‘bone'. Studies were restricted to those in the English language published between January 1996 and 30 June 2015. For the former search terms, 360 articles were found, and for the latter a further 6 articles were found. First the title, and then the abstract of each listed article was examined and only those with the following inclusion criteria were retained: human study, original study (not review), study involving comparison of a fortified food over time (not cross-sectional) when time was superior to 1 week, specific bone measurement such as bone turnover markers and/or bone mineral density, study of adults (not infants, children, adolescents, pregnancy or lactation). There were 32 distinct publications that fit the inclusion criteria. However, several articles reporting on the same cohort were published more than once. When this occurred, we grouped the articles as a single study listing all citations. Studies are grouped according to age: Table 2 for studies on young adults up to approximately age 50 years and Table 3 for studies of older adults including postmenopausal women. There were six original distinct studies on fortified foods and bone involving young adults, and 18 involving older adults (Figure 1).

## Fortification studies of young adults (<50 years)

For young adults, out of the six studies found, three involved fortifying a dairy food:^38, 39, 40^ one with inulin;^41^ two with iron;^40, 42^ three with calcium;^38, 39, 43^ and two with vitamin D.^40, 43^ Results are summarized in Table 2. The effects were not conclusive, but there is some indication that additional calcium or vitamin D^38, 39, 40^ reduced biochemical markers of bone turnover, particularly showing decreases in those markers (CTX, NTX) that reflect the bone resorption rate (Table 2).

## Fortification studies of older adults (postmenopausal women and men ⩾50 years)

There were three times more studies with adults over 50 years than below. These studies^39, 44, 45, 46, 47, 48, 49, 50, 51, 52, 53, 54, 55, 56, 57, 58, 59, 60, 61, 62, 63, 64, 65, 66, 67^ involved many combinations of nutrients added to foods (Table 3). Most studies (16 of 18) involved fortification of milk or other dairy products. In 12 studies, both vitamin D and calcium were added and in 4 trials either calcium or vitamin D (Figure 1). In these 16 studies, results were consistent in showing a reduced bone turnover with evidence of improved bone density and/or strength (Table 3). Other nutrients added to foods included magnesium, vitamin K, folic acid and isoflavones, but there were not enough replicates to provide conclusive results.

A potential concern in examining fortification studies of older adults was that several trials used only a single-arm design. However, the treatment of these single-arm design studies gave similar results—improvement in bone outcomes with vitamin D and/or calcium—to the treatment arms of the two-arm design studies (Table 3). In other studies, the fortified foods were not compared with unfortified equivalent foods. In most cases, these studies provided evidence that a fortified dairy product was efficacious in improving bone outcomes but did not provide evidence that the beneficial response was due to the specific nutrients that were added to the dairy food.

## Food vehicles for fortification

As described above, dairy was often used as the food that was fortified in the studies we found (Table 2; Table 3). Taking into account that both dietary calcium and proteins can exert positive and possibly synergistic effects in the preservation of bone integrity (see for review ref. 68), dairy is a food of interest as they contain these two nutrients in appreciable amounts, as compared with their recommended daily allowance, and can especially be useful for older adults who have low appetite and therefore are less likely to choose more than one serving of a dairy product per day. In contrast, the natural vitamin D content in dairy is far too low to be sufficient to meet the body needs, especially in countries such as Australia, France and the UK where mandatory vitamin D fortification is not practiced. In most usual western diets, unfortified foods other than dairy do not provide a sufficient supply of vitamin D to compensate for an inadequate year-round solar UVB exposure.

The fortification of milk and/or other dairy products with calcium is warranted for subjects whose daily portion size is insufficient to meet the recommended dietary allowance values as estimated by the Institute of Medicine and other national or international food agencies.^1, 69, 70^ A first considered situation was that of the low-birth weight infant fed either human milk or commercial formula that both contain insufficient quantities of calcium and phosphate.^71, 72^ Besides this very early life critical period, there are other situations when the calcium balance resulting from the usual food consumption is too low to secure the optimal needs for bone. This can be the case during childhood and adolescence to cope with the enhanced bone accrual or in postmenopausal women and elderly to prevent the accelerated bone loss. In these two physiological situations, consumption of fortified dairy products appears to be particularly appropriate for subjects who, deliberately or not, choose to limit their energy intake and ingest just one dairy serving per day.

## Biochemical markers to assess fortified food effects on bone

The main observation drawn from this scoping review is that, in the majority of the 24 trials, fortified dairy foods improved bone through reduced bone turnover. This assessment corroborates the importance of measuring circulating factors reflecting bone remodeling to test the effects of fortified foods. As recently reviewed, clinical trials aimed at testing nutritional products on bone outcomes need to use surrogate end points for assessing anti-fracture efficacy.^73^ Fragility fracture–related measurements include specific hormonal factors (PTH, IGF-I; see for reviews refs 74, 75) and bone turnover markers related either to bone formation (for example, P1NP, osteocalcin, alkaline phosphatase) or to bone resorption (for example, NTX, CTX, TRAP5b; see for reviews refs 76, 77, 78). Measurement of turnover markers within a few weeks or months after the onset of an intervention can predict the future rate of bone loss and in the long term the risk of fragility fracture.^79, 80, 81^ This prediction lies on a fundamental mechanism of bone biology: modification in remodeling is the key process on which both pharmaceutical agents and nutrients exert their anti-catabolic or anabolic action on bone structural integrity and resistance to mechanical loading.^82^

**Figure 2:**
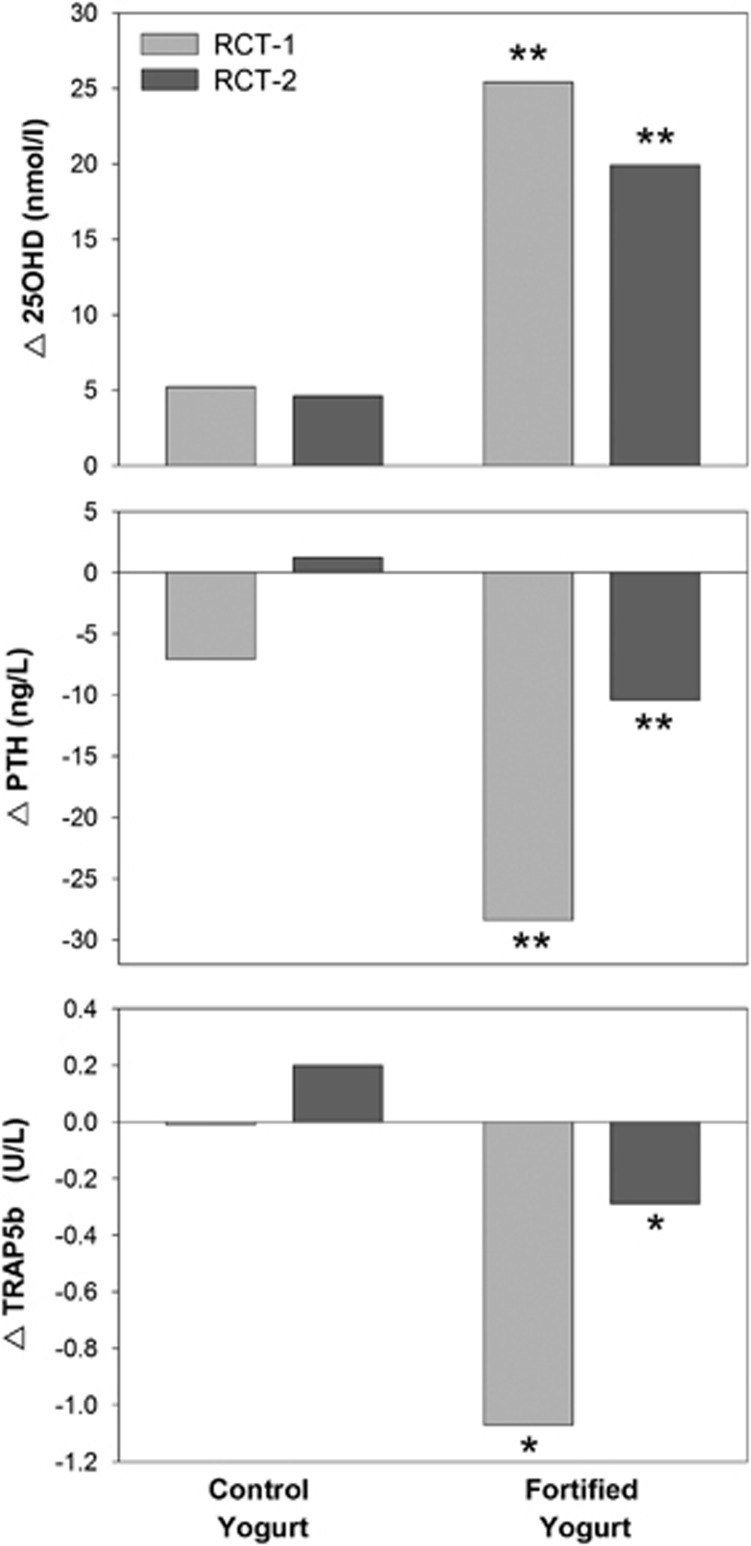
Changes (Δ) after 56 days (D56–D0) of consumption of non-fortified control or fortified yogurts in two independent double-blind randomized clinical trials. One trial (RCT-1) was carried out in women living in French (FR) nursing homes, the other (RCT-2) in women living in a Great Britain (GRB) community dwelling home. Columns represent the difference after 56 d in serum 25OHD (upper panel), PTH (middle panel) and TRAP5b (lower panel). Data are taken from two original studies published in 2013^47^ and 2015.^48^ In both trials, the differences in changes between fortified and control (non-fortified) yogurt groups were statistically significant for 25OHD, PTH and TRAP5b: **P<0.001; *P<0.0025.

In fact, use of bone turnover markers is critical to carry out food fortification studies that can be expensive because of food preparation and distribution costs, comparatively with studies testing the effects of nutrient supplements.^73^ Figure 2 illustrates that, in two independent randomized controlled trials conducted in older women, there were significantly greater effects of calcium and vitamin D-fortified yogurts vs unfortified equivalent yogurts on serum 25OHD and PTH. Furthermore, TRAP5b, a specific bone resorption marker, was consistently reduced in the two trials.

## Safety considerations

As expressed by food regulatory agencies, replacement of nutrient losses during food processing and correction of established deficiencies should guide enrichment and fortification policy, respectively (see for reviews refs 32, 83). As mentioned above, calcium and vitamin D are the two main fortificants used to improve bone health. For both micronutrients, the safety margin can be considered as relatively wide. According to the 2011 report from the Institute of Medicine (IOM), the tolerable upper levels for adults are considered to be 2000–2500 mg/d and 4000 U/d (100 μg/d) for calcium and vitamin D, respectively.^70^ Above these levels, there is a risk of adverse events such as the occurrence of hypercalcemia, hypercalciuria, nephrolithiasis, vascular and soft tissue calcification and for vitamin D even an increased risk of falls and fracture.^84^ Below these ULs, the evidence suggesting adverse cardiovascular effects of calcium supplementation is inconsistent.^85^ There is reasonable assurance that consumption of calcium and/or vitamin D-fortified foods should not result in adverse effects.

## Summary

In this scoping review, we analyzed 24 original studies in which fortified foods were evaluated for their potential beneficial effects on bone outcomes in adulthood. Calcium and vitamin D were the fortificants most often added, whereas milk and dairy-related products were the most frequently used fortified foods. Several studies rigorously compared calcium-vitamin D-fortified foods with unfortified food equivalents, in a double-blind randomized controlled design. Evidence was obtained that, in postmenopausal women and elderly, food fortification with calcium and vitamin D substantially improves vitamin D status, provides a greater prevention of secondary hyperparathyroidism and significantly reduces accelerated bone turnover. The pattern of these biochemical effects can be interpreted as beneficial to the global prevention of osteoporosis and fragility fractures with aging. However, further research is needed to examine the effects of adding more of the putative bone-healthy nutrients in one fortified food product.

## Figures and Tables

**Table 1:** EFSA positive opinions on vitamins and minerals other than calcium and vitamin D for making a bone health claim for the general population

*Nutrient*	*Approved wording in EFSA Article 113-1*	*Claimed effect*	*Conditions of use*
Vitamin K^26^	Vitamin K contributes to the maintenance of normal bone	Bone structure, bone integrity, bone calcification and bone health	At least source of=11.25 μg minimum
Magnesium^27^	Magnesium contributes to the maintenance of normal bone and teeth	Bone and teeth structure	At least source of=56.25 mg minimum
Phosphorus^28^	Phosphorus contributes to the maintenance of normal bone and teeth	Bone and teeth structure	Not inadequate intake of Phosphorus in the general EU population^a^
Manganese^29^	Manganese contributes to the maintenance of normal bone	Bone formation	Not inadequate intake of Manganese in the general EU population^a^
Zinc^30^	Zinc contributes to the maintenance of normal bone	Bone formation	Not inadequate intake of Zinc in the general EU population^a^

Abbreviations: EFSA, European Food Safety Authority; EU, European Union. Table content drawn from references 26 to 30.

Table content drawn from references 26, 27, 28, 29, 30.

a No justification to fortify foods with these nutrients according to EFSA.

**Table 2:** Scoping review of food fortification trials (2000–2015) having bone health outcomes involving young adults (<50 years) or predominantly younger adults (mean age <50 years)

*Reference (country study location)*	*Food vehicle*	*Nutrients added*	*Subjects (years)*	*Trial length*	*Bone measures*	*Results*	*Notes*
*Studies comparing fortified with unfortified foods*							
Blanco-Rojo *et al.*^42^ (ESP)	Fruit juice	Iron	Iron-deficient women (mean 25 years)	16 weeks	AKPase NTX	Not significant	Fe status improved
Dahl *et al.*^41^ (CAN)	Thickened fruit beverages	Inulin	Institutionalized young adults age (23–57 years)	3 weeks	NTX	Not significant	
Ferrar *et al.*^38^ (GBR)	Ice cream	Calcium	Women (20–39 years)	4 weeks	sCTX uNTX/Cr PTH P1NP	↑ P1NP ↓ CTX	
Kruger *et al.*^39^ (NZL)	Milk	Calcium±vitamin K	Women (20–35 years)	16 weeks	CTX P1NP OC	↓ P1NP ↓ CTX	No additive effect of vitamin K to calcium
Tapola *et al.*^43^ (FIN)	Mineral water	Folic, B6, B12, vitamin D, calcium	Men and women (mean 48 years)	8 weeks	AKPase	↑ AKPase as measure of calcium bioavailability	
*Studies where control food was not unfortified product*							
Toxqui *et al.*^40^ (ESP)	Milk	Iron±vitamin D	Iron deficiency women (18-35 y)	16 weeks	PTH P1NP NTX	↓ P1NP ↓ NTX (+Fe +D vs Fe alone)	Effect of iron on bone seen with correlation analysis

Abbreviations: AKPase, alkaline phosphatase; CAN, Canada; CTX, C-terminal telopeptide (s, serum); ESP, Spain; FIN, Finland; GBR, United Kingdom; NTX, N-terminal telopeptide (u, urine); NZL, New Zealand; OC, osteocalcin; PTH, parathyroid hormone; P1NP, Procollagen Type 1 N-Terminal Propeptide.

**Table 3:** Scoping review of food fortification trials (2000–2015) having bone health outcomes involving older adults (⩾50 years or postmenopausal women

*Reference (country study location)*	*Food vehicle*	*Nutrients added*	*Subjects*	*Trial Length*	*Bone Measures*	*Results of Treatment*	*Notes*
*Studies comparing fortified with unfortified foods*							
Adolphi *et al.*^44^ (DEU)	Fermented milk	Calcium, inulin, casein phospho-peptides	PM women	2 weeks	ALP DPY	Not significant	Milk reduced night-time bone turnover
Bonjour *et al.*^45^ (FRA)	Soft cheese	Calcium, vitamin D	Nursing home PM women	6 weeks	TRAP5b, CTX OC, PTH P1NP IGF-1	↓TRAP5b ↓ CTX ↓ PTH ↑ IGF-1	
Bonjour *et al.*^46^ (FRA)	Soft cheese	Calcium, vitamin D	PM women	6 weeks	TRAP5b, CTX OC, PTH P1NP, IGF-1	↓TRAP5b ↑ IGF-1	
Bonjour *et al.*^47^ (FRA)	Yogurt	Calcium, vitamin D	Nursing home PM women	8 weeks	TRAP5b, CTX OC, PTH P1NP, IGF-1	↓TRAP5b ↓ PTH	
Bonjour *et al.*^48^ (GBR)	Yogurt	Calcium, vitamin D	PM women	12 weeks	TRAP5b, CTX OC, PTH P1NP, IGF-1	↓TRAP5b ↓ PTH	
Brink *et al.*^49^ (FIN, FRA, ITA, GBR) a	Biscuits and bars	Isoflavones	Early PM women	1 year	BMD PYD, DPD P1NP PTH, ALP	Not significant	
Daly *et al.*^50, 51, 52^ (AUS)	Milk	Calcium, vitamin D	Nursing home men	2 years	BMD (DXA, QCT)	↑ femoral neck BMD ↑ bone strength if >62-year-old	BMD effect is sustained after 18 months follow-up
Grieger *et al.*^53^ (AUS)	Milk	Calcium, folate, vitamin D	Nursing home women	6 months	CTX P1NP PTH	Not significant	No additive effect of vitamin K to calcium
Jafari *et al.*^54^ (IRN)	Yogurt	Vitamin D	Type 2 Diabetic PM women	12 weeks	NTX PTH	↓ NTX	Dose of vitamin D was 2000 IU
Kanellakis *et al.*^55^ (GRC)	Dairy	Calcium, vitamin D,±vitamin K (K1 or K2) in 3 groups (compared to no dairy)	PM women	1 year	BMD IGF-1 DYP, PTH	↑ lumbar BMD with vitamin K	↑ total body BMD with any dairy
Kukuljan *et al.*^56, 57^ (AUS)	Milk	Calcium, vitamin D	Older men	18 months	BMD	Not significant	Men were vitamin D replete at start
*Studies where control food was not unfortified product*							
Green *et al.*^58^ (NZL)	Milk (tested against apple drink)	Magnesium	PM women	4 weeks	CTX PTH	↓ CTX	
Gui *et al.*^59^ (CHN)	Soy or cow milk (compared to no milk)	Calcium	PM women	18 months	BMD	↑ femoral neck BMD	
Kruger *et al.*^39^ (NZL)	Milk (compared to fruit drink)	Calcium, vitamin D, magnesium, zinc	PM women	4 months	CTX, P1NP OC, PTH	↓ CTX,↓P1NP ↓ OC.↓PTH	
Manios *et al.*^b^; Moschonis and Manios^b^; Moschonis *et al.*^b^; Tenta *et al.*^60, 61, 62, 63, 64^^,^^b^ (GRC)	Dairy (compared to no milk)	Calcium, vitamin D	PM women	1 year, then 1.5 years of increased vitamin D	BMD CTX PTH IGF-1	↑ total body BMD (2.5 years) ↓ PTH ↑ IGF-1	Also compared dairy to supplemental calcium and vitamin D
Palacios *et al.*^67^ (ESP)	Milk	Calcium, vitamin D – compared two doses of calcium	PM women	10 weeks	ALP P1NP PYR NTX	Higher Ca dose ↓PYR	
*Single-arm design*							
Bonjour *et al.*^65^ (FRA)	Soft cheese	Calcium, vitamin D	Nursing home PM women	4 weeks	TRAP5b, CTX OC, PTH P1NP IGF-1	↓TRAP5b ↓ CTX ↓ PTH ↑ IGF-1	Compared pre- and post-treatment
Mocanu *et al.*^66^ (ROM)	Bread	Vitamin D	Nursing home	1 year	BMD	↑ BMD *z*-scores for hip, and spine	Compared pre- and post-treatment

Abbreviations: ALP, alkaline phosphatase; AUS, Australia; BMD, bone mineral density; CAN, Canada; CHN; China; CTX, C-terminal telopeptide (s, serum); DEU, Germany; DPY, deoxypyridinoline crosslinks; ESP, Spain; FIN, Finland; FRA, France; GBR, United Kingdom; GRC, Greece; IGF-1, insulin-like growth factor-1; IRN, Iran; ITA, Italy; NTX, N-terminal telopeptide (u, urine); NZL, New Zealand; OC, osteocalcin; PM, postmenopausal; PTH, parathyroid hormone; P1NP, Procollagen Type 1N-Terminal Propeptide; PYD, pyridinoline crosslinks; ROM, Romania; TRAP5b, Tartrate-resistant acid phosphatase (TRAP) 5b.

a Multicenter PHYTOS study.

b Five reports from ‘The Postmenopausal Health Study'.
